# Theory Change in Cognitive Neurobiology: The Case of the Orbitofrontal Cortex

**DOI:** 10.1002/wcs.70003

**Published:** 2025-05-06

**Authors:** David L. Barack

**Affiliations:** ^1^ Department of Philosophy Lingnan University Hong Kong Hong Kong

**Keywords:** cognitive neuroscience, explanation, orbitofrontal cortex, theory

## Abstract

How do theories of the functions of parts of the brain change? I argue that computational hypotheses help explain the nature of theorizing in cognitive neurobiology. I will focus on the orbitofrontal cortex (OFC), a frontal region of the brain implicated in an array of cognitive functions. Different theories of OFC state different principles of OFC function and use different concepts to construct those principles. There are also differences in the patterns of use of evidence across different theories. I briefly survey several extant proposals for understanding theory change in science generally and cognitive neuroscience specifically, including paradigm shifts, tool innovation, mechanism discovery, conceptual innovation, exploratory experimentation, and changes in measurement techniques. While these extant approaches fall short at describing the nature of theory change illustrated by the case of OFC, they are compatible with my proposal that these theoretical changes and differences in the use of evidence result from different computational hypotheses about the region.

## Graphical abstract

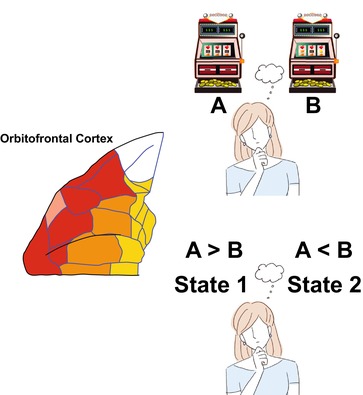

## 1 Introduction

How do theories change in cognitive neuroscience? In this essay, I will examine the nature of theorizing in cognitive neurobiology, the study and explanation of cognitive phenomena in terms of the activity of single neurons and neural populations. I focus on theories of cognitive functions for the cortex. A theory is a set of general principles that explains a body of evidence, provides understanding, and helps make predictions (Winther 2021). By evidence, I mean phenomena based on data, not the data (observations, measurements, etc.) themselves (Bogen and Woodward 1988). Phenomena are stable, repeatable features of the world that can be detected in different ways from different data; the evidence used by cognitive neurobiologists are phenomena, not data, on this construal. Herein, I will be pluralist about explanation, which may take deductive‐nomological, inductive‐statistical, causal‐mechanical, structural, topological, efficient, and other forms (Hempel and Oppenheim 1948; Salmon 1989; Craver 2007; Chirimuuta 2014, 2017; Huneman 2018). The key is that theories are essentially constituted by principles that explain bodies of evidence; different theories will explain different evidence and may do so in different ways. I will stay neutral on different philosophical approaches to theories (e.g., the syntactic, semantic, or pragmatic views (Winther 2021)), and so theories might be sets of axioms, models, or other forms. I will outline some lessons about how theories in cognitive neurobiology change regardless of the philosophical approach taken toward theories.

I will focus on the orbitofrontal cortex (OFC), a frontal region of the brain implicated in an array of cognitive functions (Rolls 2019). The case study reveals that different theories of OFC state different principles of OFC function using different concepts and appeal to different evidence, where during theory change some evidence is foregrounded while other, sometimes even contradictory, evidence is backgrounded. I propose that these changes in concepts and principles and differences in the use of evidence result from different computational hypotheses about the region, where a computational hypothesis is a hypothesis about the input to output transformations performed by some part of the brain. Computational hypotheses help explain how theories develop and change in cognitive neurobiology.

I start with a brief anatomical review of the orbitofrontal cortex (OFC). I then turn to discuss two extant theories of OFC function, value theory and task state theory. I next discuss how theorizing about the region's function turns on different principles and concepts and appeals to some evidence over others, even overlooking seemingly inconsistent evidence. I propose both facts are explained by computational hypotheses that posit different transformations over representations and appeal to some evidence over other evidence.

## 2 Current Theories of Orbitofrontal Function

**FIGURE 1:**
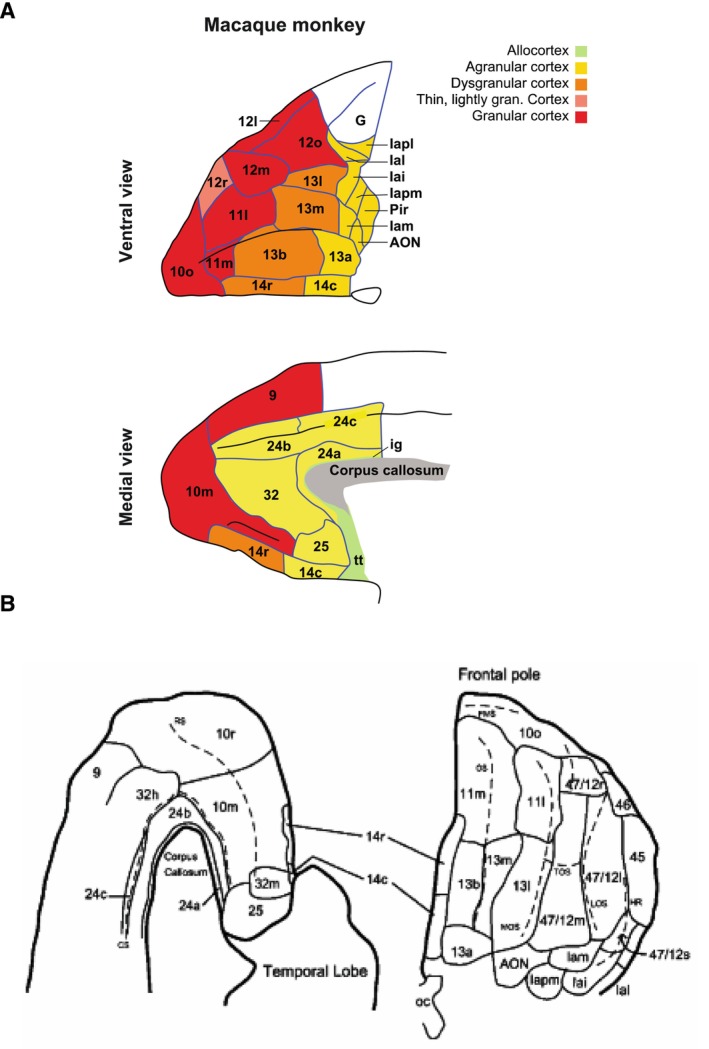
Orbitofrontal cortex. (A) Orbitofrontal cortex subregions in macaques. Top panel: Ventral surface of the brain; bottom panel: Medial surface. From Rudebeck and Izquierdo 2022. (B) Orbitofrontal cortex subregions in humans. Left panel: Medial surface of the brain; right panel: Ventral surface. From Ongur and Price 2000. OFC corresponds to areas 11, 12, 13, and 14.

The orbitofrontal cortex (OFC) is an anterior region of the prefrontal cortex that sits on the underside of the brain just behind the eyes (Figure 1). The region is traditionally identified with Brodmann Areas (BA) 11, 12, 13, and 14 (Brodmann 1909; Walker 1940). The region has extensive incoming connections from the visual, chemosensory, and tactile cortex, often reciprocated by outputs. Bidirectional connections also exist with medial temporal lobe structures and subcortical structures like the basal ganglia and thalamus. These specific input and output connections constrain theorizing about the region because, for a given proposed function, the region must be anatomically situated to receive the correct inputs and to transmit the correct outputs for the proposal. However, inter‐areal connectivity is insufficient for determining the functions of neurons in the area, as intra‐areal operations are essential to determining that functioning. After Passingham 2021, I will use the following conventions: lateral OFC (lOFC) is BA12, central OFC (cOFC) is BA11/13, and medial OFC (mOFC) is BA14.

I will now discuss two dominant theories of OFC function, value theory and task state theory, starting with value theory. The discussion of these theories will highlight aspects of theorizing in cognitive neurobiology.

### 2.1 Value Theory

The value theory of the orbitofrontal cortex states that the region represents the values of options for decision making based on sensory and non‐sensory inputs. An example is the neuroeconomic theory of value (Padoa‐Schioppa 2011). The cOFC contains neurons that represent value, such as the value of offers (Padoa‐Schioppa and Assad 2006, 2008; Padoa‐Schioppa 2009, 2013; Cai and Padoa‐Schioppa 2014, 2019; Conen and Padoa‐Schioppa 2015), that have been shown to be sufficient to generate value‐based decisions (Rustichini and Padoa‐Schioppa 2015; Padoa‐Schioppa and Conen 2017).

Evidence for the value theory reaches back to the first findings in OFC. Building on findings of gustatory and reward responses in OFC neurons (Thorpe et al. 1979, 1983; Rosenkilde et al. 1981; Rolls et al. 1989), reward processing was extensively investigated in the region. OFC neurons code the rewards associated with odors and this coding decreases after feeding the animal to satiety the juice that corresponds to the odor (Critchley and Rolls 1996; Rolls et al. 1996). These findings blossomed into a comprehensive theory of OFC function focused on cOFC (Padoa‐Schioppa 2007, 2011; Fellows 2011; Rolls 2019; Klein‐ Flügge et al. 2022; Soltani and Koechlin 2022). Single neurons in monkey cOFC signal the expected reward from upcoming outcomes (Tremblay and Schultz 2000; Wallis and Miller 2003; Roesch and Olson 2004), reward size (Bouret and Richmond 2010; O'Neill and Schultz 2015), gains and losses (Morrison and Salzman 2009; Rich and Wallis 2014), reward risk (O'Neill and Schultz 2010, 2015) and risk prediction error (O'Neill and Schultz 2013), reward probability (Kennerley and Wallis 2009a, 2009b), reward range (Conen and Padoa‐Schioppa 2019), and relative reward (Kobayashi et al. 2010; Saez et al. 2017). A similar range of choice and outcome economic variables have been found in recordings in humans (Saez et al. 2018). Finally, choices can be decoded from population activity (Rich and Wallis 2016) and values, choices, and expected outcomes are represented along orthogonal dimensions of activity in cOFC (Kimmel et al. 2020).

Causal evidence supports the value theory. Stimulation of cOFC increases choice bias in favor of the option presented without stimulation, as though value representations were disrupted (Ballesta et al. 2020). In addition, aspiration lesions to monkey OFC reveal deficits in reward‐based choice (Clark et al. 2013). Several experiments demonstrate a causal role for OFC in humans in value‐based choice as well (Bechara et al. 2000; Hornak et al. 2004; Fellows and Farah 2007; Sellitto et al. 2010; Camille et al. 2011a, 2011b; Henri‐Bhargava et al. 2012).

There are several challenges for value theories. First, lesion evidence poses problems. Animals with OFC lesions can still learn novel stimulus–reward associations no different from controls (Iversen and Mishkin 1970; Rudebeck et al. 2013), and animals who have already learned associations retain them following damage to OFC (Baxter et al. 2000; Izquierdo et al. 2004). This counts against value theories because learning or storing the value of options would seem to require representing them. Consider reversal learning failures following OFC lesions. In reversal learning, animals first learn a set of object‐reward associations, and then the associations between objects and rewards are flipped, forcing animals to adapt their behavior to the new associations. Recent lesion studies of OFC revealed no deficits in reversal learning, suggesting that storing or updating value in that task does not rely on the region (Rudebeck et al. 2013). A similar conclusion follows from devaluation studies. These studies start with a learning phase where participants learn to associate objects with preferred and non‐preferred food, followed by a devaluation phase where participants are fed the preferred food to satiety, and then a choice phase where participants freely select between objects after devaluation. Animals typically choose objects associated with the non‐satiated non‐preferred food, whereas those with cOFC lesions typically persist in selecting the preferred food despite satiation (Butter 1969; Rudebeck et al. 2013). However, there are no differences between lesioned animals and controls in the initial learning phase, which requires constructing an association between an object and a food (Rudebeck et al. 2013; Reber et al. 2017).

Besides those challenges, second, OFC neurons signal rules for flexible behavior. In a rule switching task, cOFC and lOFC neurons encode the current rule (Wallis et al. 2001). cOFC neurons also encode rules in the Wisconsin card sorting task (WCST, Sleezer et al. 2016) and stay‐shift strategies in a visuomotor conditional task (Tsujimoto et al. 2011, 2012). These findings challenge the value theory because rules reflect structure in the environment and not necessarily the value of options.

How have value theorists responded to this counterevidence? One strategy has been to refine the concepts used in the theories, such as to distinguish between value for choice and value for learning. As Padoa‐Schioppa puts it, “[i]n principle, values defined in economic choice and values defined in learning theory have nothing to do with one another. Notably, when they perform economic choices in our experiments, animals are no longer learning anything, so it is not clear why one would invoke concepts from learning theory” ((Padoa‐Schioppa and Schoenbaum 2015), 20). Here, a distinction is drawn between value for choice and value for learning, and the counterevidence is judged irrelevant because it regards the wrong kind of value. Another strategy has been to refine the proposed transformations performed by a region. For example, Murray and colleagues found that inactivation of anterior cOFC before selective satiation resulted in no impairment and after yielded reduced devaluation (i.e., the monkeys continued to select the food item that had been devalued), whereas inactivation of posterior cOFC before but not after resulted in reduced devaluation (Murray et al. 2015). Based on this finding, they distinguish between updating and retrieving value representations. A third strategy has been to search for a different theory of OFC function, which I turn to now.

### 2.2 Task State Theory

An alternative theory of OFC functioning arose in the early 2010's. OFC represents the structure of the environment, particularly the structure of tasks in which animals are engaged. This structure is often called a “cognitive map” (Tolman 1948), a map of the states and transitions between states in the environment. To illustrate, consider the decision to cross a street at an intersection (adapted from Niv 2019). As you approach the intersection, you stop on the south sidewalk and face a choice. This corresponds to the first state, S1. You can decide to “go” and cross the sidewalk, which transitions to state S2, being on the north sidewalk, with probability of 0.6 or to state S3, being in the hospital (because you have been hit by a car), with probability of 0.4. You can also choose to “wait” and let the light change, which transitions back to state S1 with unity probability. In this framework, there are states, which are typically thought of as collections of features of environments, transitions between states, and actions which take agents from one state to another with some probability.

OFC is thought to represent this structure, whether the states or the transition matrix (Wilson et al. 2014; Stalnaker et al. 2015; Niv 2019; Boorman et al. 2021). Building on non‐parametric methods in statistics (Gershman and Blei 2012), a number of quantitative proposals were developed for understanding how minds represent the structure of their environments (Courville et al. 2005; Gershman and Niv 2010; Collins and Frank 2013). These approaches were motivated by the ability of animals to represent and use this structure and by computational reinforcement learning, which posits a set of states used to help select actions to maximize reward.

A range of evidence, from both neuroimaging and neurophysiology, supports task state theory. Findings from human neuroimaging support the hypothesis that OFC represents hidden states, those that must be inferred from observable features. Schuck and colleagues tested the task state theory directly (Schuck et al. 2016). Participants had to decide whether a superimposed face/house stimulus was young or old and switch categories when signaled by changes in ages. The hidden features on the task included the previous category, the current category, and previous trial responses. Though the original presentation of task state theory in Wilson et al. 2014 tentatively identified lOFC as the location of those representations, analysis of BOLD responses revealed that only mOFC (roughly BA14) signaled all three hidden features. A number of other fMRI studies provide supporting evidence for task state theory (Chan et al. 2016; Constantinescu et al. 2016; Park et al. 2020; Boorman et al. 2021). These studies disagree about which division of OFC represents latent states, but the theory is actively being refined (Boorman et al. 2021; Gardner and Schoenbaum 2021).

Some monkey neurophysiology studies support the task state theory. For example, Wang and Hayden found that neurons in cOFC distinctly coded trial types in a described vs. experienced riskless choice task, suggesting separate representations of states (Wang and Hayden 2017). Further, rules are latent states, and so evidence for rule coding in OFC supports task state theory (Wallis et al. 2001; Buckley et al. 2009; Tsujimoto et al. 2011; Sleezer et al. 2016; Tsutsui et al. 2016). Rules can be thought of as information about which contingencies are active or as maps of the current environment's structure that can used to guide behavior. So, rule signaling by OFC neurons is straightforwardly an instance of task state theory.

Evidence for task state theory also comes from lesion studies in monkeys. Buckley and colleagues found that on the rule‐based Wisconsin card sort task, monkeys with mOFC and cOFC lesions were slower to learn rules, slower to respond, and made fewer rule switches (Buckley et al. 2009). While the monkeys were still able to learn rules and perform the task, the poorer performance provides evidence for the task state theory. The task state theory also handles the lesion findings that challenged value theory above. Consider first reversal learning deficits from lesions. There are two states in reversal learning. In the first state, option A is rewarded and option B is not. In the second state, option B is rewarded and option A is not. The initial learning of contingencies is thought to invoke slower, older learning circuits, especially in the basal ganglia, whereas fast, flexible switching between contingencies is supported by OFC. When OFC is damaged, these two states are lumped together, and the environment is treated as being in the same state after the reversal as before. Adapting to the change then requires recruitment of slower learning circuits to update the new value of options, giving rise to the failure of reversal learning (Butter 1969; Schoenbaum et al. 2000, 2003; Rudebeck and Murray 2008; Wilson et al. 2014).

Second, lesions to cOFC result in deficits during devaluation from selective satiation. Monkeys who have received lesions targeting cOFC will persevere in selecting an object associated with the preferred but sated food, whereas monkeys with lOFC lesions, in contrast, do not show failures to selectively satiate (Rudebeck et al. 2017). Task state theory analyzes selective satiation as regarding a single task state, the conditional associations between objects and food; lesioning lOFC has no impact because there is no need to form multiple task state representations or switch between them. Lesioning cOFC, in contrast, has an effect because cOFC updates task state representations including their value. Note that task state theorists do not reject a role for OFC in value‐based computations (see, e.g., Wilson et al. 2014, 274 or Schuck et al. 2016, 1407); rather, value becomes one component of task states. Without cOFC, the value of current sated contexts cannot be updated and so the monkey must rely on context‐independent preferences elsewhere in the brain to guide choices.

There are at least two concerns with task state theory. First, there are other areas implicated in the representation of task states, including the hippocampus (Bernardi et al. 2020) and the anterior cingulate cortex (Lamba et al. 2023), suggesting the task state hypothesis is too general to accurately describe OFC function. Second, some lesion evidence suggests that OFC does not represent task states (Baxter et al. 2007, 2009). Baxter and colleagues show that in a strategy switching task that required keeping track of latent strategies, monkeys with wide OFC lesions show preserved performance on the task. Monkeys earned rewards by selecting one of a pair of images drawn from two groups, one for persistent choice and one for sporadic choice. Monkeys selected persistent choice items four times in a row to get a reward, after which they selected an item from the sporadic set for a reward before returning to the persistent items. Because the current strategy and category membership for the images were not cued, monkeys had to construct and deploy representations of strategies, the latent states of the task. The performance of monkeys with extensive OFC lesions was no different from controls on this task (Baxter et al. 2007, 2009). This implies that monkeys with OFC lesions can represent latent states of the environment.

Task state theorists have responded in several ways. For example, based on value‐related findings and other evidence, Knudsen and Wallis propose that cOFC initializes or updates the values of task states (Knudsen and Wallis 2022), a partial reconciliation between the two approaches. This is a refinement of the transformation, consistent with other areas representing task states and with preserved behavior following lesions after tasks are initially learned.

In sum, there are two dominant theories of OFC function, value theory and task state theory. The debate about the functions of the region is lively and ongoing, and new experimental results and theories, including other reconciliations (see, e.g, Wikenheiser and Schoenbaum 2016, Klein‐ Flügge et al. 2022, Knudsen and Wallis 2022, Rudebeck and Izquierdo 2022), are being proposed. For my purposes, the details above are sufficient to turn to discuss some implications for theorizing in cognitive neurobiology.

## 3 Theorizing in Cognitive Neurobiology

Having painted a picture of recent theories for the orbitofrontal cortex (OFC), I turn to draw some philosophical lessons about theorizing in cognitive neurobiology. A satisfactory account of theory change should explain two facts illustrated by the case study. First, theories change in the principles and concepts that describe a brain area's function in the explanation of some behavior. Second, during theory change, many findings regarding brain regions are ignored while others are more salient. An account of theorizing should explain both aspects of theory change. I will explain these aspects by appealing to the role of computational hypotheses in cognitive neurobiological theorizing. While I am ultimately a pluralist about the drives behind theory change and my proposal is consistent with many approaches canvassed below, many extant approaches overlook the role of computational hypotheses for understanding how theories develop and change in cognitive neurobiology.

Theory change is characterized by appeal to different principles and concepts to explain the evidence for neurocognitive function. The principles and concepts used to describe the function of some brain regions are parts of theories, and those principles and concepts change, in the sense that different ones are used, when theories do. I include a range of ways that concepts can change, from wholesale replacement, such as can occur when one theory replaces another, to refinement, as when a concept develops within a theory. Take value theory. Central to value theory are concepts like *reward magnitude* and *reward probability* (concepts will be italicized herein). These concepts are used to state the function of OFC in value‐based choice; specifically, the product of reward magnitude and probability is the expected reward from selecting an option (Bernoulli 1738). That formula is one of the principles of value theory (Glimcher 2011), used to explain observed neural activity, deficits, and so forth. Task state theory, in contrast, appeals to a different set of principles using different concepts. Specifically, *task state* is a concept drawn from computational reinforcement learning (Sutton and Barto 1998). Task state theory conceptualizes these states as categories, and a set of states and the transitions between them are inferred from observations and rewards, described using a range of formalisms for belief updating, like non‐parametric Bayesian methods (Ferguson 1973; Antoniak 1974; Gershman and Blei 2012). These principles are then used to explain the evidence. The move from value theory to task state theory would be more like a replacement than a refinement. In contrast, distinguishing *choice value* from *learning value* as Padoa‐Schioppa does within the context of value theory would be refinement.

Different theories also exhibit different patterns of evidence use. This second fact is also illustrated by the case of OFC. Consider task state theory. The theory's development was partly inspired by some outlier findings that go back as far as the origins of value. Specifically, the first lesion findings for reversal learning and selective satiation deficits go back to the inception of research in the region (Butter et al. 1963; Butter 1969; Iversen and Mishkin 1970). Both of those effects are evidence against value theory, because learning the initial conditional associations in reversal learning, and the formation of the initial conditional associations as well as unconditioned preferences in selective satiation, all rely on value computations preserved in lesioned animals. And yet, value theorists cite the contradictory evidence. For example, Padoa‐Schioppa, whose neuroeconomic theory focuses on cOFC, notes that selective satiation “…is specifically impaired after lesions of [mOFC]…. In contrast, [selective satiation] is not affected by lesions of [cOFC]…. Second, …recent studies using excitotoxic agents… found that [cOFC] lesions alone do not affect reversal learning” (Padoa‐Schioppa and Conen 2017, 738–739). Lesions to cOFC that fail to affect selective satiation should be evidence against the neuroeconomic theory because that satiation relies on devaluing the sated option. And failures to disrupt reversal learning should also count against the theory, because that learning is based on changes in the value of options. Despite the contrary indications of these lesion findings, the studies are positively cited in the foregoing. The reason is that Padoa‐Schioppa draws a distinction between *choice value* and *learning value*; the point is that we want an account of theory change that can accommodate these kinds of theoretical moves. A similar story can be told for the task state theory with regard to the lesion findings from Baxter and colleagues (Baxter et al. 2007, 2009). Animals with OFC lesions exhibit preserved performance despite the need to represent latent states, the strategies required for the task. So, like the case for value theory, for the same overall body of evidence, some evidence is cited in support and other evidence is ignored, with changes in this pattern of evidence use across different theories.

What explains these two facts about theory change in cognitive neurobiology? Focus in the philosophy of science on theory change is often restricted to physics, such as Kuhn's classic account which relies on dominant paradigms and anomalies (Kuhn 2012). Here, I will discuss recent proposals from the philosophy of biology or neurobiology.

Some philosophers propose that tool development drives theorizing in neuroscience (Dyson 2000, 2012; Bickle et al. 2022; Haueis 2023). Bickle has stated that “…in “wetlab” neurobiology, new tool development *drives everything else*. …Theory in wetlab neurobiology is dependent upon the development and ingenious use of new research tools. …Theoretical progress in this …science …is secondary to and dependent upon new tool development, both temporally and epistemically” (Bickle 2022, 13–14, italics in original). Insofar as cognitive neurobiology is wetlab neurobiology, the case study of the OFC is evidence against Bickle's hypothesis. The task state theory's principles (such as Bayesian updating of latent states) and concepts (such as *task state*) did not arise out of the development and use of some new tool; rather, it was the convergence of well‐known, outstanding counter‐evidence (the lesion studies reviewed above), a set of mathematical formalisms (Gershman and Blei 2012), basic experimental science initially conducted in rodents (Schoenbaum et al. 2000), and modeling (Courville et al. 2006; Wilson et al. 2014) that gave rise to the task state hypothesis. And value theory also did not arise due to tool development. Rather, increasing experimental evidence for a wide range of activity in OFC (see the long account in Rolls 2019) beyond gustatory responses contributed to its development. Neither did either theory result from novel uses of old tools or from novel analysis techniques (Haueis 2023).

Mechanism discovery has been a recent area of philosophical research underlying theory change (Darden 2002; Bechtel 2008; Bechtel and Richardson 2010; Craver and Darden 2013; Kästner and Haueis 2021; Haueis and Kästner 2022). However, whether cognitive neuroscientists describe mechanisms is debated (Craver 2007; Chirimuuta 2014; Ross 2015; Huneman 2018; Barack 2021). Furthermore, the methods outlined for discovering mechanisms, including decomposition and localization, forward/backward chaining, or modular subassembly (Bechtel and Richardson 2010; Craver and Darden 2013) do not obviously explain the case of theory change for OFC.

Consider changes in principles and concepts. The shift from *value* to *task states* and *belief updating* is not the result of finding modules, understood as sets of entities and activities organized in a fashion to produce some outcome, that are assembled as part of a larger mechanism (i.e., modular subassembly). Forward/backward chaining “…involves first learning something about the mechanism or one of its components and then using that knowledge to make inferences about what came before it or what is likely to come after it” (Craver and Darden 2013, 104–105). The changes in principles and concepts don't involve this kind of identification of mechanism parts and then inferring precursor and successor stages.

Next consider Bechtel and Richardson (2010), who identify four constraints on theory development: psychological constraints (“heuristic strategies”), phenomenological regularities (“behavioral capacities of the system”), operational constraints (“experimental procedures and models”), and physical constraints (which “…limit the range of… component functions”) (Bechtel and Richardson 2010, 234). Changes in OFC theorizing are motivated by phenomenological regularities, such as discovering differences in behavior following lesions, though seemingly not by operational or physical constraints. Two key heuristics are decomposition and localization. In decomposition and localization, scientists decompose the overall capacity of the system to perform a task into component functions and then localize those functions to parts. Countervailing evidence provides a reason to refine proposed decompositions, such as distinguishing different senses of value or shifting to the task state theory. These changes are consistent with the heuristics described by Bechtel and Richardson, who say, “[i]f there is a failure of localization and decomposition, we can make adjustments at either level, or both” (Bechtel and Richardson 2010, 148). While changes in OFC theories can be cast as changes in decomposition, the nature of the changes remains undescribed on their view. Extra explanatory machinery is required.

Mechanists are concerned with functions for the entities and activities described by neuroscientists. Craver outlines three ways that function plays a role in discovering mechanisms (Craver 2013). Functions are important to etiological explanations, which “highlight the pathway connecting relevant set‐up conditions in the past, through intermediate stages of activity, to the item to be explained” (Craver 2013, 145). But principles and concepts don't feature in theorizing as changes in etiological explanations. Second, the description of functions is “…contentful… to the extent that they explicitly make claims about how an item is situated in its causal context” (Craver 2013, 154). The changes in principles and concepts for OFC theories, however, does not mainly contribute to differences in describing the causal context of a mechanism. Rather, they describe the region's functioning, not the causal context of that functioning. Finally, functions also appear in mechanisms as “…a mapping from inputs to outputs in conformity with a rule” (Craver 2013, 149), which “…define the relevant input‐output relationships that the internal mechanism must be capable of performing” (Craver 2013, 151). This statement accurately describes how functions are conceived in cognitive neurobiology, but Craver outlines only two roles for these input–output mappings. First, “such abstract description affords the scientist descriptive leverage over the messy details of the constitutive mechanism that produces the… function” (Craver 2013, 150), and second, they are “…important for characterizing the phenomena for which one will seek constitutive explanations” (Craver 2013, 150). The role of input–output mappings is in describing and constraining the mechanistic explanations that neurobiologists devise, and so a specification of the transformation is a precursor to mechanistic explanations. But since I am concerned with explaining changes in theories of those transformations, which comes before mechanistic explanation on Craver's view, the two aspects of theory change in OFC can't consist in describing and constraining mechanistic explanation. Instead, different input–output relations are specified by the principles and concepts specified by the theory, such as Padoa‐Schioppa's proposal to distinguish *choice value* from *learning value* with different computations for each, which can then go on to constrain mechanism.

Unlike changes in principles and concepts, which are partly covered by the accounts provided by Craver and by Bechtel and Richardson, the changes in the pattern of evidence use are harder to explain by these various mechanism discovery methods.

Another strand in philosophical thought on theorizing is that theory development results from conceptual change (Thagard 1993) or innovation (Nersessian 2010; Feest 2012). However, the concepts used in cognitive neurobiological theorizing are consistent across different theories of brain regions, and changes in concepts need not drive theory development, as the case of OFC illustrates. *Reward*, *value*, *task state*, and other concepts in the investigation of OFC function were typically defined before the start of the investigation and are often constant across theories. *Value* plays a role in the task state theory as one feature of states, for example, and that concept is intended to be identical to the one that appears in value theories. Concepts do differentiate, such as the distinction between *choice value* and *learning value*, but conceptual change and innovation alone do not explain the different patterns of evidence use. Again, a further factor is needed.

Exploratory experiments have also been utilized to explain theory development in biology (Burian 1997; Steinle 1997; Haueis 2023). Haueis defines three steps of concept formation through exploratory experimentation: meaning, significance, and reference (Haueis 2023, 356). But the development of task state theory for OFC was not the result of exploratory concept formation; specifically, the meaning step, where neuroscientists “[u]se experimental conditions to form operational definition” (Haueis 2023, 356), is violated, as task state theorists did not operationally define task states based on some experimental condition. An initial presentation of the task state theory (Wilson et al. 2014), for example, used computational reinforcement learning to specify the meaning of task states. The changes in principles and concepts during OFC theory change, then, does not match exploratory concept formation. Once again, patterns of evidence use are also unexplained.

Finally, measurement is often an impetus for theorizing and scientific change (Chang 2004). But changes in tools for or methods of measurement did not drive theorizing in the case of OFC. While each theory does inspire novel paradigms, both also interpret evidence from some of the same paradigms using the same tools to measure OFC activity. This, then, leaves changes in principles and concepts unexplained. Further, changes in measurement can notexplain the patterns of evidence use, which reflect both the collection of new data and the interpretation of old (and *a fortiori* no change in measurement).

Shifts in paradigms, tool innovation, mechanism discovery, conceptual change and innovation, exploratory experimentation, or changes in measurement techniques may explain some theorizing in cognitive neurobiology. But the case study of OFC illustrates that some additional factor is needed to explain some aspects of theory change in cognitive neuroscience.

I suggest that computational hypotheses drive theory change by determining both the concepts that inform the principles used to state generalizations and the relevance of evidence, explaining patterns of evidence use. Herein, computation is understood as input representations that are transformed into output representations that help give rise to behavior. A computational hypothesis states that for some behavior, some part of the brain has the function of transforming inputs into outputs. Changes in principles are changes in computational hypotheses, with distinct transformations and/or distinct input or output representations, and are expressed with different concepts. Further, differences in hypothesized transformations or representations partly determine the different patterns of use of evidence, because the inputs and outputs are specified in the context of some behavior that is taken to require the computation. So, some neurophysiological evidence is more relevant and others less because the data were collected during behavior that does not require some transformation, inputs, or output.

The development of value theory supports this interpretation. The theory grew out of gustatory responses, where OFC neurons effectively act as something like food detectors, signaling the presence and identity of aliments. The discovery of selective satiation effects in neural coding spurred further theorizing, resulting in the development of value theory. A detector model where the neuron signals the presence and/or quantity of a food ultimately changed into an economic model where neurons compute the expected value of choices. Taking the product of reward magnitude and reward probability transforms those two input representations into the output representation of the expected value. This is a change in both outputs (e.g., a scaled signal for food into one for expected reward) and transformations (e.g., some weight on the size and other characteristics of foods into the sum of the possible reward outcomes and their probabilities).

Neurobiologists also assess the relevance of evidence by thinking about what computations might be required for some behavior. These differences in computation explain the pattern of evidence use. Behavior that seems to require a computation similar to the computation under study is deemed relevant, and the salience of that evidence is increased, whereas behavior deemed irrelevant results in decreased salience for that evidence. This (dis)similarity is assessed at the level of the transformation or the inputs or outputs. The appeal to different computations for different behaviors helps explain the patterns of evidence use because different contexts require different computations, with OFC capable of performing both. Brain regions are multifunctional, which does not undermine function ascription (Barack 2025).

To illustrate, recall that value theory posits that neurons in OFC represent value, but that lesions revealed deficits during reversal learning or devaluation. In response to this counterevidence, Padoa‐Schioppa suggests a refinement in the concept of *value* used in value theory to distinguish between *choice value* and *learning value*. Evidence from learning‐related studies is less relevant than from choice‐related ones because the differences in paradigms require different computations, with distinct variables and transformations to explain the observed behavior. This reasoning illustrates how computational hypotheses make some evidence more relevant and other evidence less so.

A similar lesson holds for task state theory, but regarding the refinement of the computation instead of the concepts used in a theory. In response to counterevidence that OFC lesions do not impair performance on tasks that require task state representations and that other brain regions represent task states, Knudsen and Wallis distinguished between representing task states and initializing or updating values over them, and posited that OFC is required only to initialize or update those values (Knudsen and Wallis 2022). However, this novel reconciliation between the two approaches ignores the OFC lesion evidence above of preserved initial value learning. In response, OFC theorists could then draw a further distinction between *value initialization* and *value updating*.

Other philosophers have advanced similar ideas regarding computational hypotheses. Irvine states that computational templates (Humphreys 2002, 2004), “abstract syntactic schemata, such as sets of equations or computational methods that are interpreted to generate models of particular phenomena” (Irvine 2016, 152), inform computational models and underlie cognitive neuroscientific theorizing (Irvine 2016). Computational templates are uninterpreted formalisms that are transferred from one field to another and then interpreted via a “correction set” “based on relevant background empirical, theoretical, and practical knowledge” (Irvine 2016, 153). Templates are sometimes used in this way. But some cognitive neuroscientific theorizing imports interpreted models, such as expected value models from economics for the value theory, while other theorizing starts with *de novo* interpreted formalism, such as hidden task state inferences for the task state theory. The transformation in a computational hypothesis often also needs further formalization to be a model of neural activity and so applicable to neural evidence. In short, computational hypotheses either guide construction of or inform selection of computational models that are used to test theories. It is the computational hypotheses that explain theory change.

Computation may be special to systems like the brain. Unlike other complex systems like ecosystems or many‐body problems in physics, brains are thought to be computational systems. Because they are computational systems, an additional type of hypothesis, the computational hypothesis, can be brought to bear. These computational hypotheses express principles couched in terms of some concepts, and changes in computational hypotheses are changes in theoretical principles and concepts. Computational hypotheses also explain patterns of evidence use, making some evidence more relevant and some less. Importantly, this role for computational hypotheses is consistent with many approaches to theory change canvassed above; my proposal is meant as an addition that can explain these two prominent features of theory change in cognitive neurobiology, not as a replacement or to be in opposition to those other proposals. For example, my proposal can be viewed as complementing Craver's description of the roles for input–output transformations and as a computational specification of how Bechtel and Richardson's decomposition works in cognitive neurobiology. In addition, I am not claiming that all theory change is computational, and the extant approaches surveyed above may be sufficient to account for some theory change. What the focus on computational hypotheses adds is an explanation of some theory change in neurobiology that regards the special role that computation plays in theorizing about neural function. On my proposal, then, besides paradigms, tools, concepts, mechanisms, and measurements, computations drive theorizing.

## 4 Conclusions

In this essay, I used the orbitofrontal cortex to highlight theory change in cognitive neurobiology. The orbitofrontal cortex is an evolutionarily sophisticated part of the prefrontal cortex that has been the focus of investigation for over 50 years. After presenting some theories for orbitofrontal functioning, including that the region constructs representations of value for decision making and that the region represents the task structure of the environment, I turned to discuss how theories have changed. Supported by a consideration of how neurobiologists conceptualized their own theories of the orbitofrontal cortex, I argued that differences in computational hypotheses about the region express different principles and concepts and help explain patterns of evidence use in cognitive neurobiological theorizing.

## Author Contributions

**David L. Barack:** conceptualization (equal), data curation (equal), formal analysis (equal), funding acquisition (equal), investigation (equal), methodology (equal), project administration (equal).

## Conflicts of Interest

The author declares no conflicts of interest.

## Related WIREs Articles

Functionalism as a philosophical theory of the cognitive sciences.

## Data Availability

Data sharing is not applicable to this article as no new data were created or analyzed in this study.
